# Eucalyptus derived heteroatom-doped hierarchical porous carbons as electrode materials in supercapacitors

**DOI:** 10.1038/s41598-020-71649-9

**Published:** 2020-09-03

**Authors:** Yanliang Wen, Liang Chi, Karolina Wenelska, Xin Wen, Xuecheng Chen, Ewa Mijowska

**Affiliations:** 1 Department of Nanomaterials Physicochemistry, Faculty of Chemical Technology and Engineering, West Pomeranian University of Technology, Szczecin, Piastów Ave. 42, 71-065 Szczecin, Poland; 2 Shanghai Institute of Space Power-Sources (SISP), 2965 Dongchuan Road, Minhang District, Shanghai, China

**Keywords:** Electrochemistry, Materials for energy and catalysis, Porous materials, Energy storage

## Abstract

Carbon-based supercapacitors have aroused ever-increasing attention in the energy storage field due to high conductivity, chemical stability, and large surface area of the investigated carbon active materials. Herein, eucalyptus-derived nitrogen/oxygen doped hierarchical porous carbons (NHPCs) are prepared by the synergistic action of the ZnCl_2_ activation and the NH_4_Cl blowing. They feature superiorities such as high specific surface area, rational porosity, and sufficient N/O doping. These excellent physicochemical characteristics endow them excellent electrochemical performances in supercapacitors: 359 F g^−1^ at 0.5 A g^−1^ in a three-electrode system and 234 F g^−1^ at 0.5 A g^−1^ in a two-electrode system, and a high energy density of 48 Wh kg^−1^ at a power density of 750 W kg^−1^ accompanied by high durability of 92% capacitance retention through 10,000 cycles test at a high current density of 10 A g^−1^ in an organic electrolyte. This low-cost and facile strategy provides a novel route to transform biomass into high value-added electrode materials in energy storage fields.

## Introduction

The potential energy crisis caused by the rapid consumption of the traditional and nonrenewable fossil fuels has aroused increasing concerns^1^. Current social developments request strategies to provide novel, sustainable, and sufficient energy supply while ensuring low environmental pollution^2^. Supercapacitors can meet these demands in view of their features including low maintenance cost, high power density, rapid charge/discharge rate (can be fully charged/discharged in seconds), and outstanding long-cycle stability, which are at the forefront in the field of energy storage^2,3^. Generally, the performance of supercapacitors depends on the physicochemical properties of the electrode materials^4,5^.

Carbonaceous materials such as activated carbons^5^, carbon dots^6^, carbon nanotubes^7,8^, carbide-derived carbons^9^, carbon nanofibers^10^, carbon nanocages^11^, graphene materials^12^, hierarchical porous carbon (HPCs)^4,13–18^, and heteroatom-doped carbons (HDCs)^1,3,6,19,20^, have been regarded as the most attractive electrode materials for supercapacitors because of their high conductivity, large surface area, tailorable surface chemistries, and low cost^19^. Among these carbon materials, HPCs and HDCs are the most promising. In comparison to traditional carbon materials, the HPCs have attracted great research interest owing to the unique nanoporous hierarchy, which have developed wide potential applications e.g. in electrochemical capacitors, lithium-ion batteries, solar cells, hydrogen storage systems, photonic materials, fuel cells, sorbents for toxic gas separation and so on^4,13–18,21–23^. The well-defined pore dimension and topology can offer minimized diffusive resistance to mass transport by macropores and high surface area for active site dispersion over the micro and/or mesopores^21^. Many raw materials can be served as the precursors for the synthesis of HPCs, such as alcohols, carbohydrates, polysaccharides, lignocellulose sources, pitches, phenolic resins and organic polymers, which are origins from the nonrenewable oil^23^. As a promising renewable resource, biomass offers an attractive raw material due to natural abundance and low cost^21,23^. Concerning supercapacitors, the multimodal pores in HPCs are attractive to the electrode materials, in which the micropores can absorb the electrolyte ions to accumulate high capacitances, meso-/macropores are used for the ion storage, diffusion, and transport to give high rate capability^4,16,17^. Up to now, various methods have been applied to fabricate HPCs, including template method^15,18^, activation^24–26^, and their combination^27,28^. But strong corrosive etching agents (HF, strong base, HNO_3_, H_3_BO_3_) must be used. Zinc chloride (ZnCl_2_) has been demonstrated as the activation agent to synthesize HPCs with reasonable porosity and high yield for supercapacitors^20,29,30^. ZnCl_2_ acts as a dehydrating agent allowing more carbon to remain in the structure^29,30^. Recently, a kind of highly efficient and facile chemical blowing strategy has drawn significant attention to prepare HPCs^31–34^. On the other hand, the presence of heteroatoms in HDCs can introduce extra pseudo-capacitances and accelerate the interaction of electrode-electrolyte^35^. Usually, the researchers used in-situ doping or post-treatment approaches to prepare HDCs^36^. The disadvantages of these multi-step methods are the extra addition of heteroatom-contained organic chemicals, which usually are toxic and harmful. Additionally, these strategies always showed low productivity and high expense resulted from the harsh conditions and tedious process^37^. Considering sustainability and environmental concerns, biomass-derived HDCs have been widely investigated due to its natural advantages such as abundant sources, naturally doped property, and low cost^38–42^. Eucalyptus has attracted plenty of interest in the preparation of electrode materials for supercapacitors^1,43–48^. It is one of the 25 most common tree genera among the ten most common species reported by 88 countries in our world owing to its rapid growth rate, wide availability, and renewable advantage^48,49^. However, previous works used precursors of eucalyptus leaves or bulk eucalyptus grandis wood to synthesize carbon products through activation/functionalization processes with expensive and strong corrosive reagents (NaOH, KOH, HNO_3_), which are far from mass-production, sustainability, and environmental protection. Therefore, a facile and green strategy is of great significance to prepare heteroatom-doped HPCs used in energy storage applications^16,50^.

Therefore, in this contribution, a combined strategy of ZnCl_2_ activation (instead of KOH) and NH_4_Cl chemical blowing is used to synthesize nitrogen-doped HPCs (NHPCs) from cheap, abundant, and natural eucalyptus powder (EP). The current work reports the characterization and electrochemical behavior of the carbon materials followed by the proposed mechanism for the excellent performance. The effect of the mass ratio of ZnCl_2_ to EP on the pore development of NHPCs and the corresponding performance in supercapacitors has been systematically studied. When the mass ratio of ZnCl_2_ to EP reaches 5, the NHPC-5 shows the superior performance with a high gravimetric specific capacitance. Additionally, its excellent electrochemical durability (94.4% in an aqueous electrolyte of 6 M KOH and 91.7% in an organic electrolyte) has been recorded through a long-term test of 10,000 cycles at a high charge/discharge of 10 A g^−1^. We believe that this efficient and simple approach will attract great interest in the preparation of NHPCs-based electrode materials in energy storage areas.

## Results and discussion

**Figure 1:**
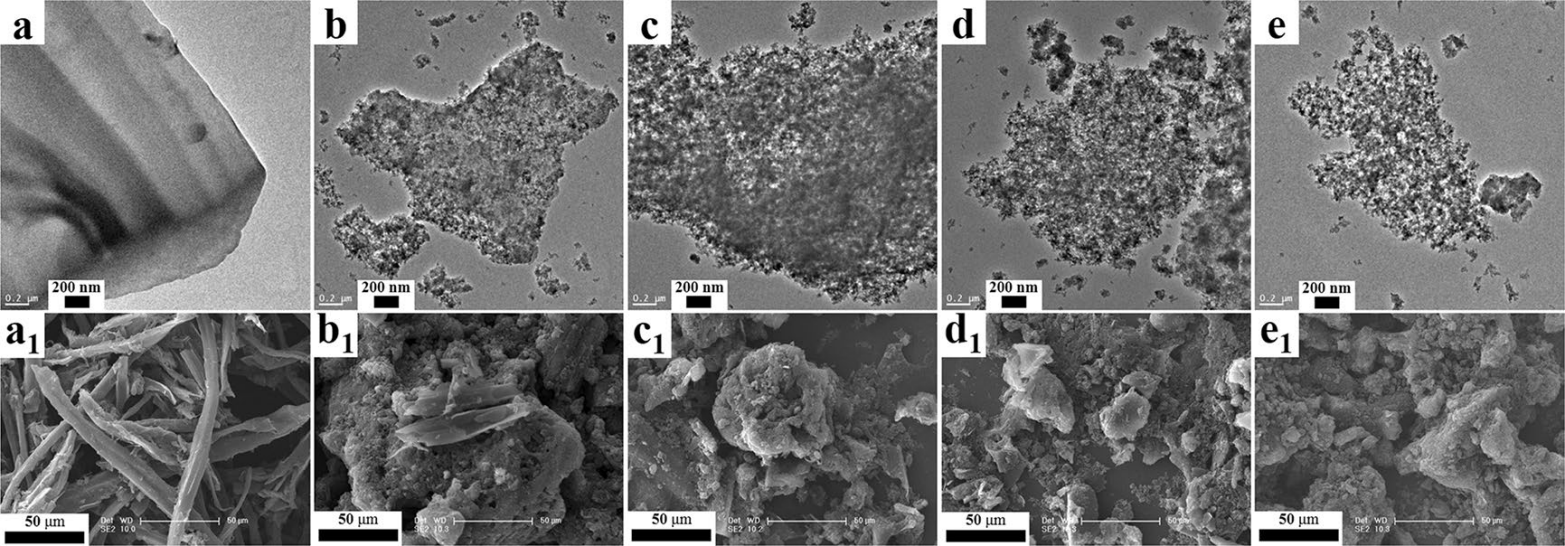
TEM images with the scale bar of 200 nm: (**a**) EC, (**b**) NHPC-1, (**c**) NHPC-3, (**d**) NHPC-5, and (**e**) NHPC-7; The corresponding SEM images with the scale bar of 50 μm: (**a**_**1**_) EC, (**b**_**1**_) NHPC-1, (**c**_**1**_) NHPC-3, (**d**_**1**_) NHPC-5, and (**e**_**1**_) NHPC-7.

The microstructure and morphology of carbon materials were observed by TEM and SEM microscopy (Fig. 1). The structures with irregular shape and broad length distribution are detected in EC, which is obtained directly from raw eucalyptus powder (EP) **(**Fig. 1a,a_1_). The morphology of the carbonaceous materials prepared with different ratios of ZnCl_2_ after carbonization and activation in nitrogen changes significantly. TEM (Fig. 1a–e) and SEM (Fig. 1a_1_–e_1_) images of these samples reveal porous and loose structures. Especially, NHPC-5 shows the uniform porous architecture (Fig. 1d,d_1_), which would be beneficial to improve the specific capacitance of supercapacitors. When the mass ratio of ZnCl_2_ to EP increases to 7, NHPC-7 displays the most amorphous structure (Fig. 1e,e_1_), indicating the worse conductivity and graphitization, which would hamper the performance in supercapacitors.

**Figure 2:**
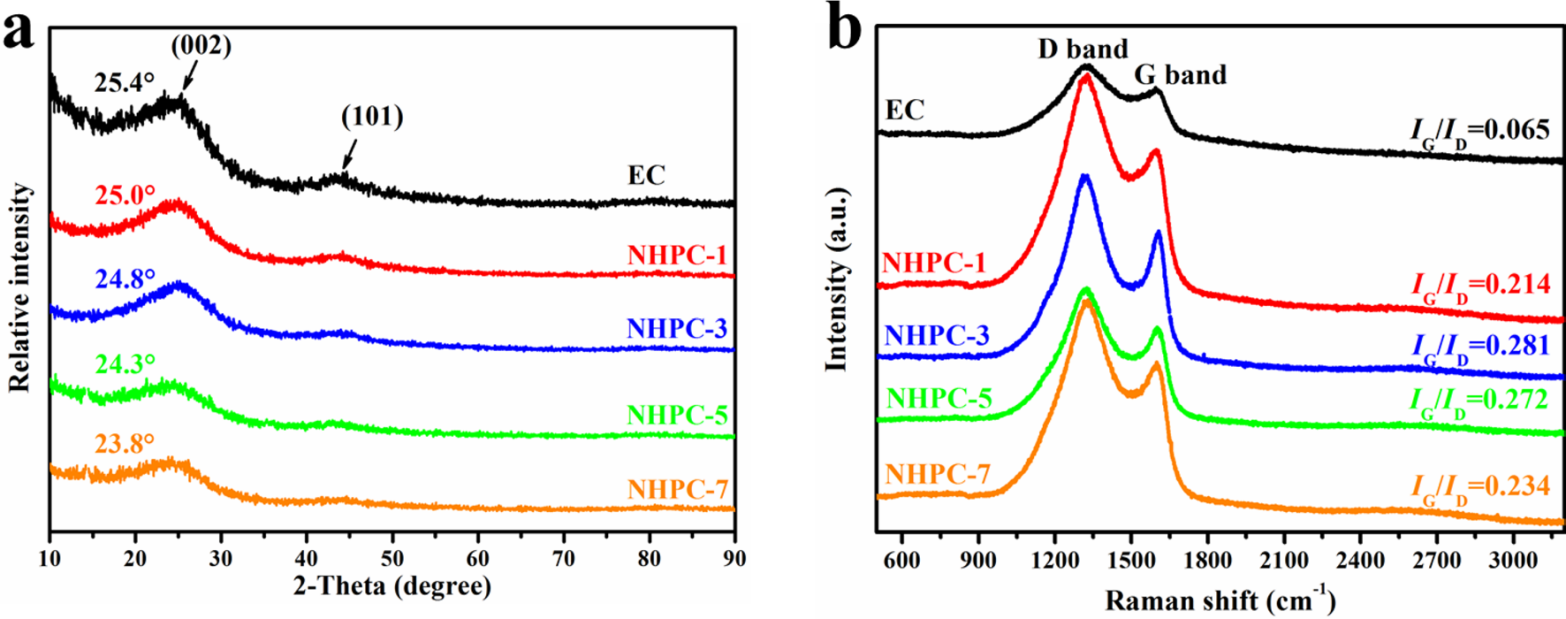
XRD patterns (**a**) and Raman spectra (**b**) of carbon materials.

In order to investigate their structure in greater detail, XRD was employed. The patterns of each sample (Fig. 2a) contain two characteristic peaks of carbon materials. After heat treatment, the XRD pattern exhibits a diffraction peak at about 24°, which corresponds to the reflection in the (002) plane of aromatic layers, and a peak barely visible at about 42°, corresponding to the reflection in the (101). The intensities of the (002) and (101) diffraction peaks are comparable for all samples. The broad peak with the weak intensity of diffraction indicates the amorphous feature of carbon materials. To further confirm the carbon structure of the sample Raman spectroscopy was used. The Raman spectra of the samples (Fig. 2b) show characteristic D (1,326 cm^−1^) and G (1,597 cm^−1^) peaks, indicating the successful formation of sp^3^-type disordered carbon and sp^2^-type graphitic carbon after thermal annealing at a high temperature of 850 °C. The ratio of the intensity of G/D peaks is to determine the relative amount of defects in the carbon structure. Here, with the increase in the mass ratio of ZnCl_2_ to EP, the G/D increases what indicates more and more sp^2^ bonds are broken which in turn means that there are more sp^3^ bonds are formed. The ratio of G/D is the highest in pristine material (EC), which can explain the formation of the more porous defected structure after chemical treatment^51^.

**Figure 3:**
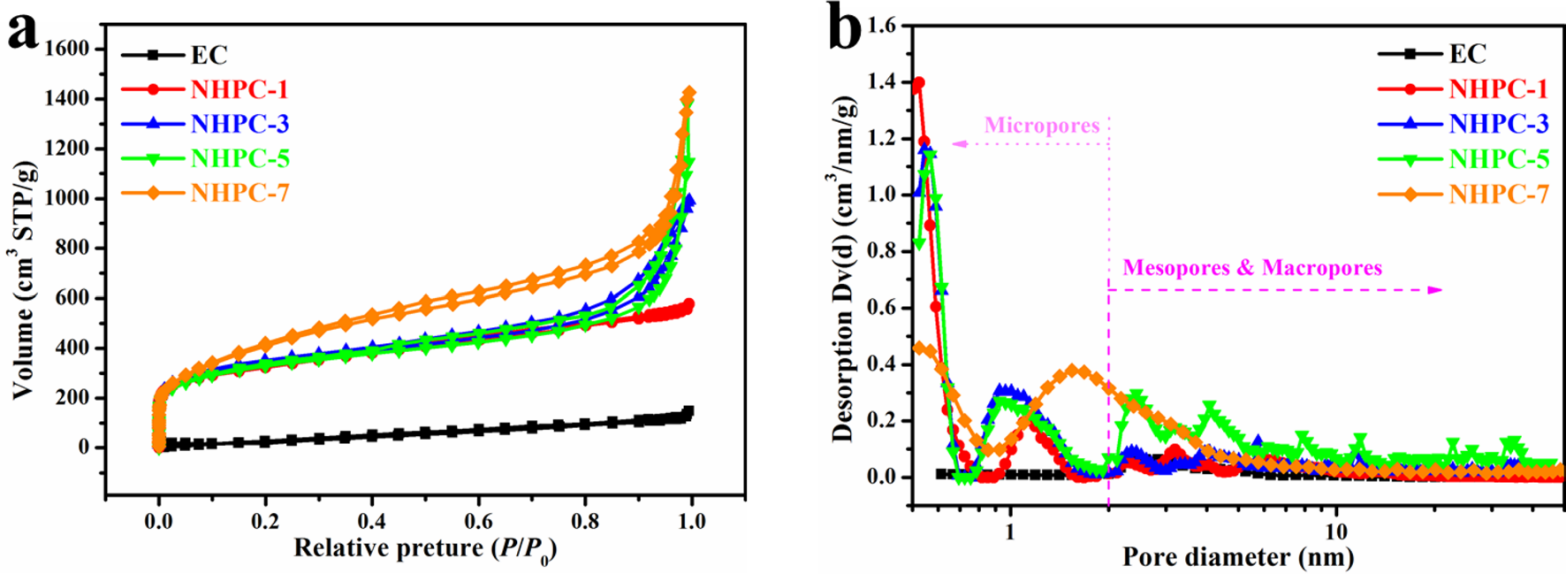
N_2_ adsorption–desorption isotherms (**a**) and pore size distribution (**b**) of carbon materials.

The nitrogen adsorption–desorption isotherms of the materials prepared at various ratios are shown in Fig. 3a. According to the refinement of original IUPAC classification^40^, EC shows the type III isotherm without any hysteresis loop, suggesting a nonporous or macroporous solid. It is consistent with the observation in Fig. 1a,a1. The MHPC-1 possesses the type I (a) isotherm shape, meaning that it is microporous material. The NHPC-*x* displays the composite of type I and II with the H4 loop. It manifests that the NHPC-3, NHPC-5, and NHPC-7 are micro-mesoporous carbons. The BET results from the nitrogen adsorption/desorption isotherms are summarized in Table 1. The EC has a specific surface area of 168 m^2^ g^−1^ and a relatively low pore volume of 0.23 cm^3^ g^−1^. The specific surface area is 1,052.9; 1,174.2; 1,331.9, 1,492.6 m^2^ g^−1^ and pore volume is 0.89; 1.53; 1.83, 1.32 cm^3^ g^−1^ for NHPC-1; NHPC-3; NHPC-5, and NHPC-7, respectively. Figure 3b presents the pore size distribution of the synthesized materials. The pore size distribution is one of the most important factors characterizing materials used for energy storage. The nonlocal density functional theory (NLDFT) was used to determine pore size distribution and can analyze from micropore to mesopore size distribution as a unified theory, which is one big advantage in comparison to other pore size analysis theories. For all carbonized samples, two small peaks in the micropore area are observed. Only one sample (NHPC-5) presents the intensive peaks in meso- and macropores range. Here, the diameter of the pores is at ~ 0.6, 0.9, 2.4, 4.1, 7.8, 11.8, 22.8, and 35.1 nm. The presence of the pores in this range is crucial in order to enhance the energy density of carbon materials applied as active material in supercapacitors.

**Table 1:** The textural parameters of carbon materials derived from the N_2_ adsorption–desorption measurements.

Sample	*S*_BET_ (m^2^/g)	*S*_micro_ (m^2^/g)	*S*_meso_ (m^2^/g)	*V*_total_ (cm^3^/g)	*D*_AV_ (nm)	Microporous fraction (%)
EC	168.0	0	168.0	0.23	3.06	0
NHPC-1	1,052.9	534.8	518.1	0.89	3.06	50.8
NHPC-3	1,174.2	545	629.2	1.53	3.83	46.4
NHPC-5	1,331.9	550.4	781.5	1.83	3.83	41.3
NHPC-7	1,492.6	614.5	878.1	2.32	3.41	41.2

**Figure 4:**
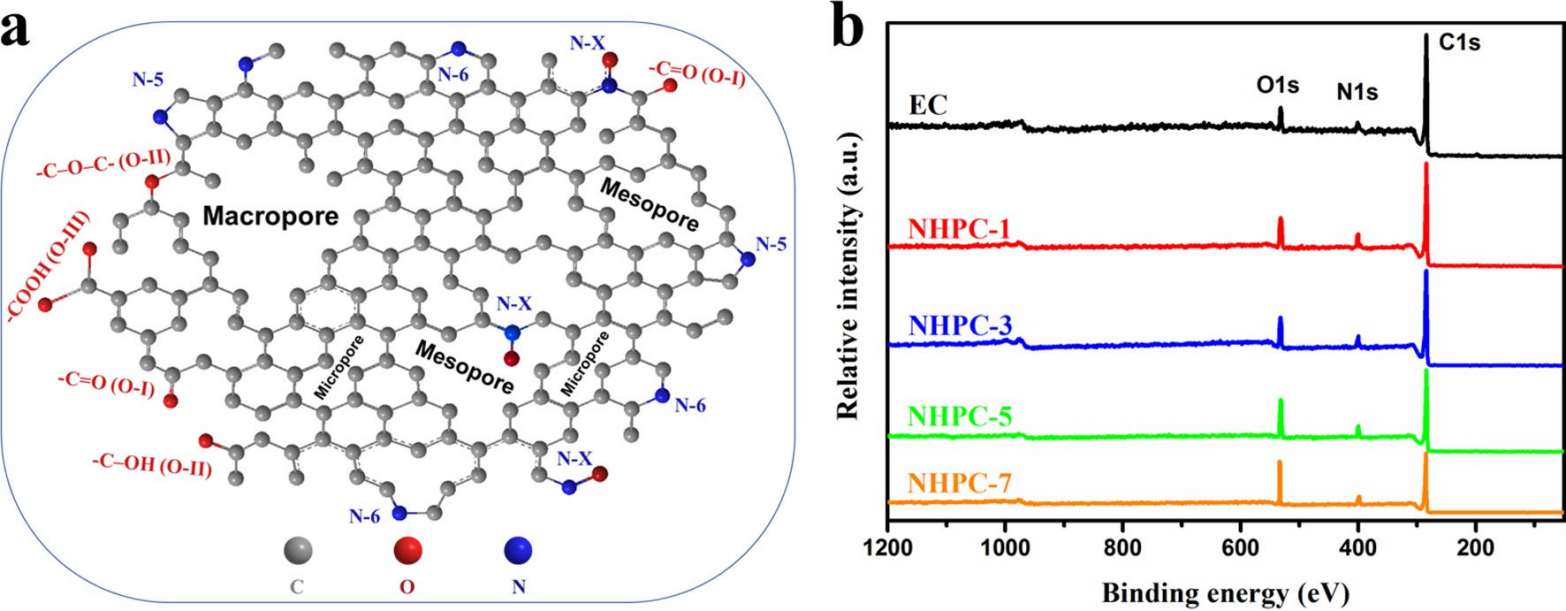
A schematic diagram (**a**) of the heteroatom-doped porous structure of NHPC-5 and XPS survey spectra (**b**) in as-prepared samples.

A schematic diagram of the heteroatom-doped hierarchical porous structure of NHPC-5 is presented in Fig. 4a. The surface elemental composition of carbons was assessed by XPS analysis. The carbon (~ 285 eV), oxygen (~ 530 eV), and nitrogen (~ 400 eV) signals are exhibited in the XPS survey spectra (Fig. 4b), which means that these carbons contain nitrogen- and oxygen-containing functional groups. These heteroatoms can give high capacitance through extra pseudo-capacitance and improve the wettability of electrode in supercapacitors^52^.

High-resolution XPS of all samples is shown in Fig. S1. It displays the atomic biding states of the C1s, O1s, and N1s elements. The detailed peak assignment and the content of each curve-fitted peak are summarized in Table S1. The C1s scans can be derived into 4 peaks with different contributions. The positions of the peaks are centered at ~ 284.2 eV (graphitic carbon, sp^2^ hybridized carbon), 285.0 eV (–C–O /–C–N come from phenol or ether), 286.2 eV (–C=O/–C=N, carbonyl or amide groups), and 288.8 eV (− COOH, ester or carboxylic groups)^53^, respectively (Figs. S1a_1_–a_5_). The percentage of sp^2^ hybridized carbon decreases with an increase in the mass of ZnCl_2_, suggesting the activation role of ZnCl_2_. Meanwhile, NHPC-5 has the highest fraction of the carbonyl or amide groups (Table S1). In the case of oxygen, the core-level XPS spectra displays three types of oxygen-containing functionalities (Fig. S1b_1_–b_5_): O–I (–C=O, quinone type groups at 530.3 ± 0.4 eV), O–II (–C–OH/C–O–C, phenol/ether groups at 532.2 ± 0.2 eV) and O–III (–COOH, chemisorbed oxygen or carboxylic groups at 533.5 ± 0.2 eV)^54^. It is reported that the O–I (C=O groups) are electrochemically active to introduce extra pseudo-capacitance^3^. Apparently, NHPC-5 possesses the highest content of O-I species, which is consistent with the results of C1s. Thus, this can be responsible for the superior capacitive performance of NHPC-5 based supercapacitors. In the case of N1s, four peaks with different chemical environments can be deconvoluted (Fig. S1c_1_–c_5_): pyridinic N (N–6), pyrrolic N (N–5), quaternary N (N-Q), and pyridinic-N-oxide (N-X)^53,55,56^.

The contents of carbon, oxygen, and nitrogen for all 5 samples are listed in Table 2. Basing on the fractions of four types of nitrogen species (Table S1), the actual contents of these nitrogen-containing groups are calculated and shown in Table 2. The nitrogen content of NHPC-5 (5.9%) is not the highest, but it has the highest content of N-6 plus N-5. It is reported that the negatively charged N-6 and N-5 can interact with the electrolyte ions to give extra pseudo-capacitance, while the N-Q and N-X can improve electron transportation through the carbon matrixes^54,55^. The chemical formulas of C, O, and N in NHPC-5 are illustrated in Fig. 4a. Combining with the results of N_2_ adsorption–desorption, NHPC-5 possessing a hierarchical porous structure coupled with the high content of heteroatom doping (oxygen and nitrogen), may show superior performance in supercapacitors.

**Table 2:** Element composition and the detailed content of the four curve-fitted peaks of N1s by XPS.

Sample	Element content			The specific content of N1s (%)	
	C (%)	N (%)	O (%)	N-6 + N-5	N-Q + N-X
EC	94.1	2.5	3.4	2.5	–
NHPC-1	89.4	6.2	4.4	6.2	–
NHPC-3	88.7	6.0	5.3	4.1	1.0
NHPC-5	84.7	5.9	9.4	4.4	1.5
NHPC-7	81.5	5.2	13.3	3.9	1.3

**Figure 5:**
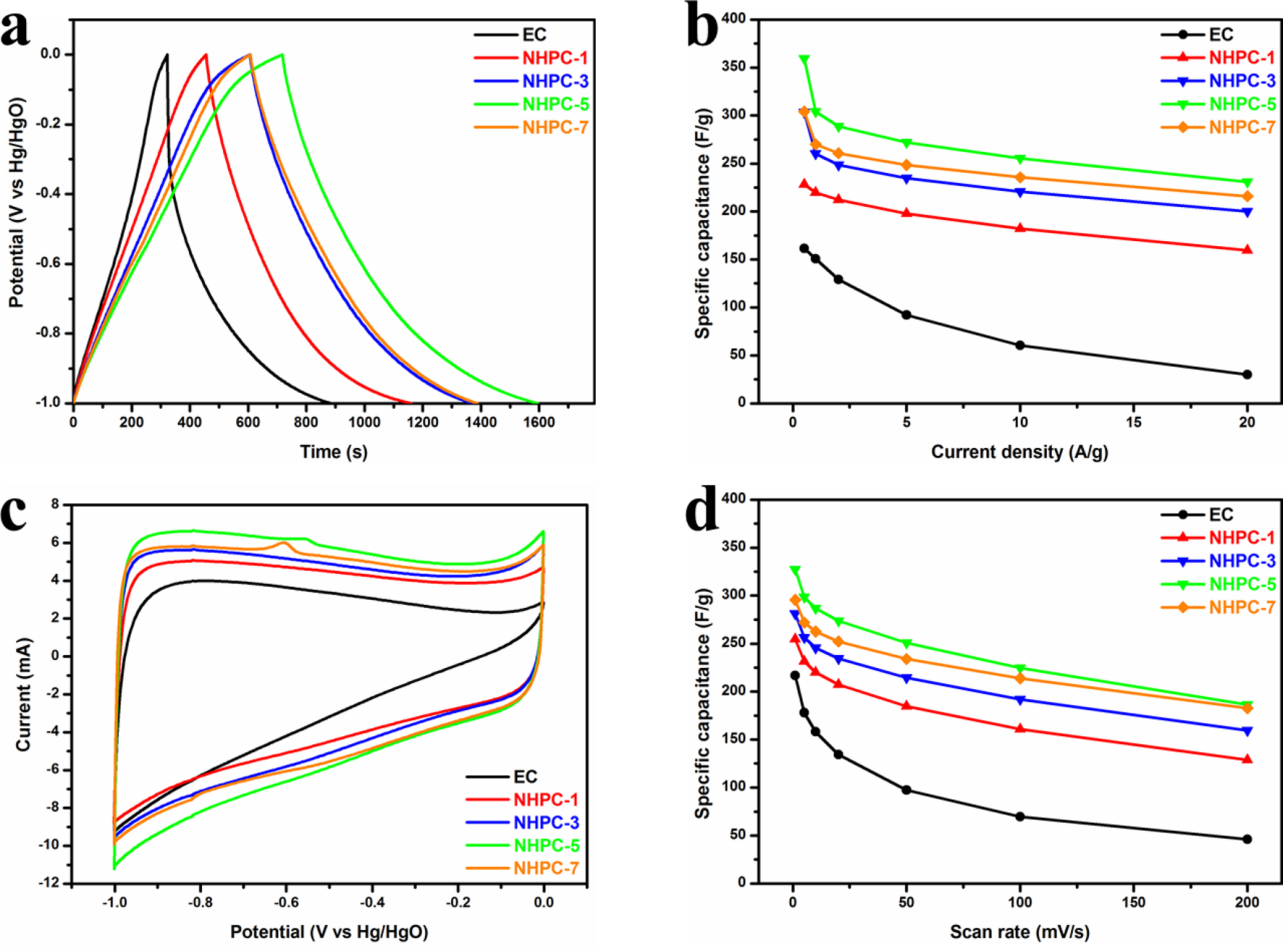
Electrochemical performances in 6 M KOH in a three-electrode system: (**a**) GCD curves at the current density of 0.5 A g^−1^; (**b**) GCD derived specific capacitances *vs* different current density; (**c**) CV curves at the scan rate of 10 mV s^−1^; and (**d**) the specific capacitances calculated from CV curves over the whole scan rate.

The as-prepared porous nitrogen-rich carbons have been evaluated in supercapacitors as the N-species can give the pseudo-capacitive performance. The GCD curves were recorded under various current densities from 0.5 to 20 A g^−1^ (Fig. S2). The comparison of GCD curves at 0.5 A g^−1^ is depicted in Fig. 5a. NHPC-5 has the longest discharge time, indicating the largest capacitance value at this test condition. For all current densities, the capacitive performance is summarized as Fig. 5b. The specific capacitances of NHPC-*x* show few degradations when increasing the current density. And obviously, the NHPC-5 is the best one. Notably, the specific capacitances of NHPC-5 are superior to that of the commercial activated carbon (TF-B520 with a specific surface area of 2000 ± 100 m^2^ g^−1^) based supercapacitors over the whole current density range in the charging-discharging measurements^57^. CV measurement was carried out at scan rates from 1 to 200 mV s^−1^ (Fig. S3). The humps that appeared in CV curves result from redox reactions caused by the doped heteroatoms in the carbon matrix, which can significantly enhance the specific capacitance of the carbon materials in supercapacitors. Figure 5c exhibits the CV curves for all samples at 10 mV s^−1^. It shows nearly rectangular shapes for NHPC-*x* at the high scan rate, manifesting the desired capacitive behaviors of supercapacitors. The largest integral area of NHPC-5 signifies the highest specific capacitance. The detailed capacitances calculated from all CV curves are shown in Fig. 5d. Comparing to the EC, the capacitive performances of NHPC-*x* in supercapacitors are significantly improved. It is consistent with the results obtained from the GCD curves (Fig. 5a,b), in which the NHPC-5 electrode exhibits superior performance. This is ascribed to the synergistic effect of NH_4_Cl blowing and ZnCl_2_ activation. As a result, hierarchical porous structures and the high specific surface area must be responsible for these excellent electrochemical performances.

**Figure 6:**
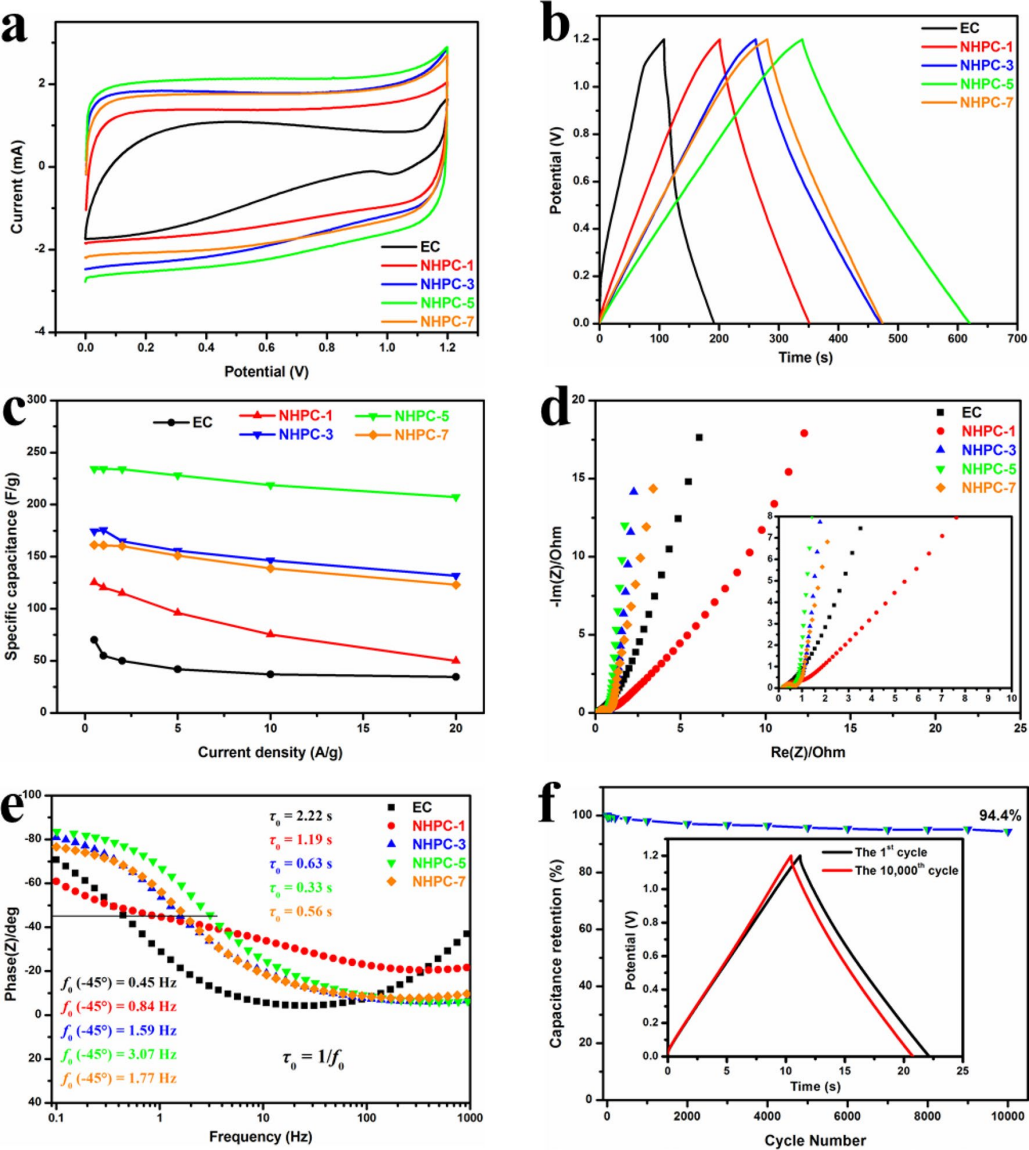
Electrochemical performances in 6 M KOH in a two-electrode configuration: (**a**) CV curves at the scan rate of 10 mV s^−1^; (**b**) GCD curves at the current density of 0.5 A g^−1^; (**c**) GCD derived specific capacitances over the whole current density; (**d**) Nyquist plots; (**e**) Bode plots; and (**f**) cyclic stability at 10 A/g for 10,000 cycles for NHPC-5 based electrode in supercapacitors.

Considering practical application, a symmetric two-electrode configuration was occupied to estimate the capacitive behaviors of the resultant samples in 6 M KOH electrolyte. In terms of all carbon materials, CV and GCD measurements have been conducted and displayed in Figs. S4 and S5 For the sake of simplicity. CV curves (at 10 mV s^−1^) with quasi-rectangular shapes are shown as Fig. 6a. The GCD curves at 0.5 A g^−1^ reveal nearly symmetrical equicrural triangles with an unobservable IR drop (Fig. 6b), which further demonstrate the ideal capacitive behaviors in supercapacitors. Figure 6c shows that the specific capacitive performance of NHPC-5 is very stable with an increase in the current density from 0.5 to 20 A g^−1^. It follows that NHPC-5 shows the best performance, both from CV and GCD curves. The corresponding specific capacitance is 234 F g^−1^ (0.5 A g^−1^) and 264 (1 mV s^−1^). It is higher than that of well-known commercial activated carbon YP17 (Kuraray Chemical, 158 F g^−1^)^58^.

The Nyquist plots based on the EIS data are displayed in Fig. 6d. NHPC-5 electrode shows very low combined resistance between the electrolytes, electrode, current collectors, which is represented by the intercept at the beginning of the semicircle^59^. It also has the smallest diameter of the semicircle in the high-frequency region, which means the highest conductivity of the electrode and the easiest diffusion of the electrolyte ions to the electrode materials. This is usually defined as the interfacial charge transfer resistance. The perpendicular line of the NHPC-5 electrode at the low-frequency zone corresponds to the fast ion diffusion process. Thus, the NHPC-5 electrode possesses the lowest equivalent series resistance (ESR) (the partial enlarged drawing of Fig. 6d). The relaxation time constant τ_0_ is another important parameter that can be investigated using the EIS technique^60^. The phase angle *vs* applied frequency is drawn as the Bode plots in Fig. 6e. The characteristic frequency *f*_0_ of NHPC-5 is 3.07 Hz when the phase angle is − 45°. The corresponding time constant τ_0_ (τ_0_ = 1/*f*_0_) is 0.33 s, which is much shorter than that of previous carbon-based supercapacitors^61^. The change of impedance is in accordance with the above-presented electrochemical performances. In summary, the NHPC-5 electrode provides a shorten ion diffusion distance and easy access of electrolyte ions to both the surface and the interior of the electrode. This can be attributed to the unique hierarchical porous structure. When it comes to the electrochemical stability (Fig. 6f), NHPC-5 electrode also shows the excellent performance of 94.4% capacitance retention of the initial value after 10,000 cycles test at an ultrahigh current density of 10 A g^−1^. The inset in Fig. 6f reveals just a little difference between the 1st and the 10,000th cycles, demonstrating the high durability of the NHPC-5 based supercapacitor. It is also the best durability among the studied samples (see Fig. 6f, Fig. S6**)**. All the detailed electrochemical performances of samples through both two- and three-electrode system in 6 M KOH is listed as Table S2. Although the capacitances are lower than that of metal oxide-based supercapacitors^62,63^, these N-doped carbon electrodes show higher cycle life with few capacitance decay, which can promote their practical application in energy-storage field. The Ragone plot shows the relation of energy and power density (Fig. S7). Owing to the high specific capacitance, the NHPC-5 electrode exhibits an energy density of 11.7 Wh kg^−1^ at the power density of 150 W kg^−1^, which is better than described in previous reports (Fig. S7).

In order to verify the synergistic function of the ZnCl_2_ activation and the NH_4_Cl blowing, the carbon material of NPC-5 prepared at the mass ratio of 1:5 was fabricated into a symmetric supercapacitor with 6 M KOH electrolyte. The CV curves and GCD curves, as well as the capacitive comparison with the NHPC-5 electrode, are shown in Fig. S8. It demonstrates that the performance of NHPC-5 is much better than that of the sample without NH_4_Cl blowing, indicating added value by the synergistic action of ZnCl_2_ activation and the NH_4_Cl blowing.

**Figure 7:**
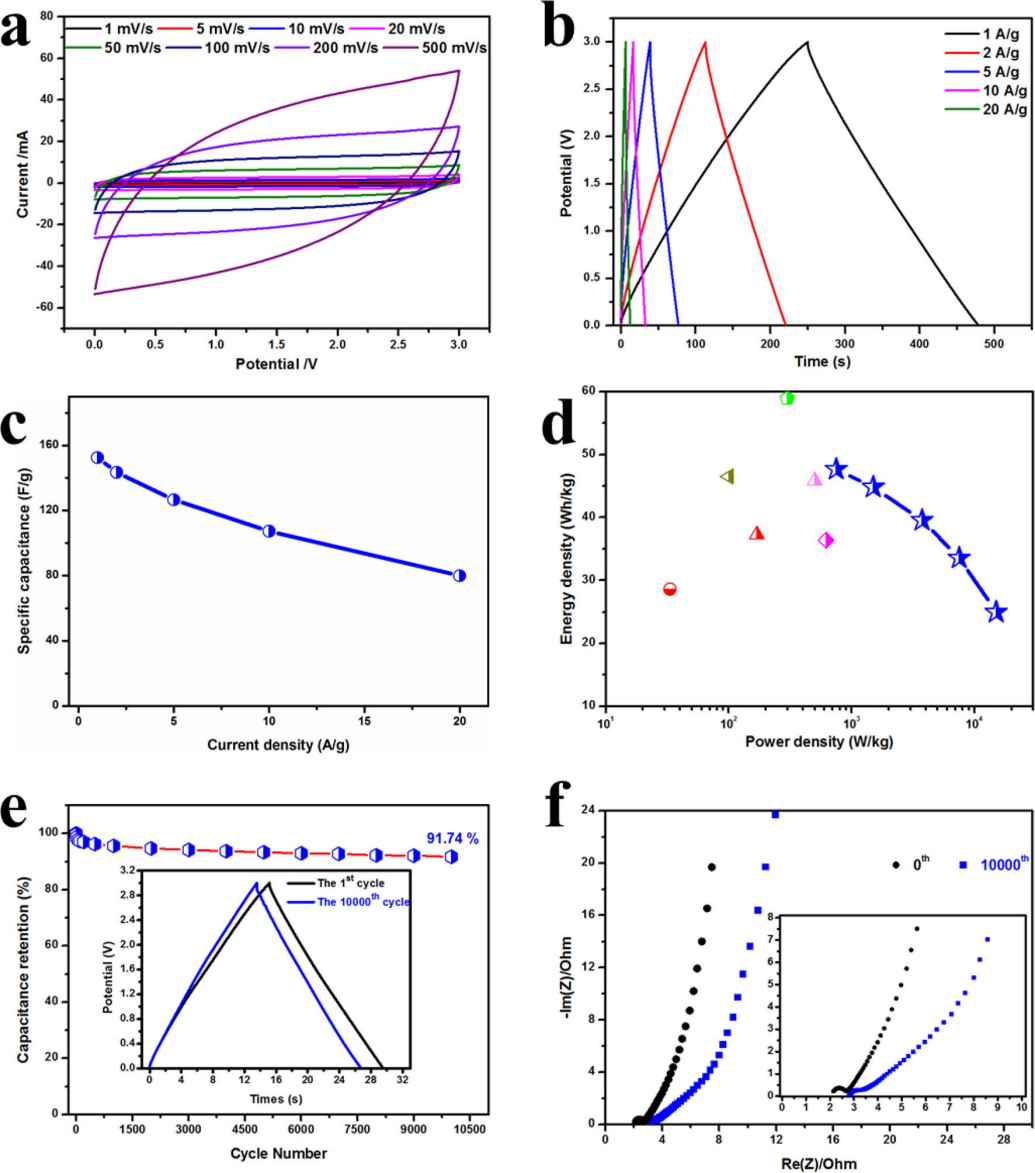
Supercapacitor performance of NHPC-5 in an organic electrolyte: (**a**) CV curves, (**b**) GCD curves, (**c**) specific capacitances calculated from the GCD curves; (**d**) Ragone plots of the power density *vs* energy density; (**e**) electrochemical durability was tested through 10,000 cycles at a high charge–discharge current density of 10 A g^−1^; (**f**) Nyquist plots in comparison of before and after long-cycle test.

The energy density *E* (Wh/kg), (the energy accumulated in an electrochemical capacitor), is a very important parameter of supercapacitors for the real applications. According to Eq. (5), *E* is proportional to the square of the voltage window and the specific capacitance. So, in order to achieve a high energy density, expanding the potential window of the used electrolyte is the first choice that should be considered. In contrast to the narrow operating voltage of aqueous electrolytes, the organic electrolyte of 1 M [BMIm]BF_4_/AN can be operated at a wide potential window of 3 V on account of its unique properties^64–66^. And it was used to achieve possible higher energy and power densities of NHPC-5 based supercapacitors. The CV profiles show the quasi-rectangular shape even at the ultrahigh scan rate of 200 mV s^−1^ (Fig. 7a). The nearly symmetric triangles without obvious IR drops are shown in the GCD curves (Fig. 7b). Both indicate the typical capacitive properties of NHPC-5 for supercapacitors. The calculated specific capacitance is 137 F g^−1^ from GCD measurements at 1 A g^−1^ coupled with a high rate performance of 72 F g^−1^ when the current density increases to 20 A g^−1^ in this organic electrolyte (Fig. 7c). The Ragone plot of power density versus energy density is shown in Fig. 7d. Benefiting by the wide working voltage and the high specific capacitance, the organic supercapacitor delivers a maximum energy density of up to 48 Wh kg^−1^ with a power density of 750 W kg^−1^ at 1 A g^−1^, which is much better than that of most reported biomass-derived carbons^4,65,67–70^. Owing to the good rate capability, when the current density increases to 20 A g^−1^, the energy density maintains 25 Wh kg^−1^ at an ultrahigh power density of 15 kW kg^−1^, which is still superior to that in the aqueous electrolyte of 6 M KOH (12 Wh kg^−1^). It is noted that commercial supercapacitor usually has a relatively low energy density (< 5Wh kg^−1^), which is usually lower than 10% of the NHPC-5 based device^71,72^.

Furthermore, a 10,000 cycles test at a current density of 10 A g^−1^ was performed to study the stability of this device. It shows a good cycling electrochemical stability of 92% capacitance retention after the long-term performance (Fig. 7e). The finite distortion of the shapes between the 1st and 10,000th further demonstrate the good stability of NHPC-5 based supercapacitor (the inset in Fig. 7e). The Nyquist plots also show that the curve does not change two much after the 10,000 charging-discharging cycles (Fig. 7f). The ESR after the long-cycle performance is still very small (~ 3.3 Ω). These results prove that NHPC-5 possesses a high potential to be used as the electrode material in supercapacitors with high energy density and excellent durability for practical usage. It is attributed to the synergistic effects of the relatively high surface area, the unique hierarchical porous structure, and the adequate N and O doping.

## Conclusions

In summary, a combined method of ZnCl_2_ activation and NH_4_Cl blowing has been applied to transform the cheap, abundant, and natural EP powder into the heteroatom doped carbons with a hierarchical porous architecture, high surface area and high content of heteroatom (nitrogen and oxygen) doping. The as-synthesized carbon material of NHPC-5 displays outstanding performances with aqueous electrolyte in supercapacitors: 359 F g^−1^ at 0.5 A g^−1^ (three-electrode) and 234 F g^−1^ at 0.5 A g^−1^ (two-electrode). When it is used in an organic electrolyte, it demonstrates a high energy density of 48 Wh kg^−1^ at a power density of 750 W kg^−1^ accompanied by high capacitance retention of 92% after a 10,000 cycles test at a 10 A g^−1^. This efficient and low-cost combined strategy will open a new avenue for transforming biomass into high value-added nanocarbon materials used as electrode active materials in supercapacitors. We believe this work would stimulate plenty of interest in the preparation of heteroatom-doped hierarchical porous carbon materials for energy storage areas.

## Experimental section

### Materials

Eucalyptus powder (EP) was purchased from a local supermarket (Carrefour, Szczecin, Poland) a few months ago. ZnCl_2_, NH_4_Cl and HCl were collected from Sigma-Aldrich Chemical Co. All chemicals of analytical grade occupied in experiments were used directly without any further purification.

### Synthesis of nitrogen-doped hierarchical porous carbons (NHPCs)

EP (1 g) was dispersed in 1 M NH_4_Cl (19 mL) solution with continually magnetic stirring and ultra-sonication. After a homogeneously dispersed phase was obtained, a certain amount of ZnCl_2_ was added to the above mixture. The mixture was stirred at a rapid speed at least for 3 h to form EP emulsion. Then the emulsion was heated at 60 °C to evaporate the moisture. Finally, the dried mixture was obtained. The as-prepared material was put into a corundum boat and carbonized at 850 °C for 2 h under N_2_ atmosphere at the flow rate of 100 mL min^−1^ in a horizontal quartz tube. The heating rate is 3 °C min^−1^ coupled with a cooling rate of 4 °C min^−1^. Subsequently, the common hydrochloric acid was used to purify the residue. Finally, a series of NHPC-*x* were obtained, where the *x* (1, 3, 5 and 7) represents the mass ratio of ZnCl_2_ to EP. The carbon prepared without any ZnCl_2_ was noted as EC.

### Materials characterization

The morphology was observed through a field-emission scanning electron microscopy (SEM). The images of transmission electron microscopy (TEM) were acquired by a transmission electron microscope (Tecnai F30, Thermo Fisher Scientific, Waltham, MA, USA). The accelerating voltage was 200 kV. X-ray diffraction (XRD) patterns were recorded to investigate the crystal composition of the products. The X’Pert Philips Diffractometer (Cu Kα, λ = 1.5406 Å) was from Almelo, Holland. Raman spectra were obtained to evaluate the degree of graphitization of the samples through a Raman spectrometer (λ = 785 nm, Renishaw, New Mills Wotton-under-Edge, UK). The thermal stability and purity of the samples were studied by thermogravimetric analysis (TGA) through the DTA-Q600 SDT TA Instrument (New Castle, DE, USA). The tested ambiance was in a flow (100 mL min^−1^) of air (79% N_2_ and 21% O_2_) with a rate of 10 °C min^−1^ to 900 °C. X-ray photoelectron spectroscopy (XPS) was carried out by a VG ESCALAB MK II spectrometer (Al Kα radiation 10.0 kV, 10 mA). The specific surface areas (SSA) and the pore size distribution (PSD) was obtained through the nitrogen adsorption/desorption measurement on an ASAP 2460 micromeritics analyzer. The Brunaue-Emmertt-Teller (BET) was used to calculate the SSA_BET_ and the non-local density functional theory (NLDFT) was applied to investigate the PSD of the materials. The degassing process was conducted at 200 °C for 12 h before measurements.

### Electrochemical tests

All the electrochemical measurements were tested by the EC-LAB VMP3 multichannel generators (BioLogic Science Instruments, France) through both two- and three-electrode systems. In the three-electrode system, the working electrode was fabricated by the following steps: the active material, carbon black and poly(tetrafluoroethylene) (PTFE) were mixed in a mortar at a mass ratio of 8:1:1. And it was manually grounded with acetone to form a well-mixed slurry. Then the slurry was coated on nickel foam with a diameter of 1 cm followed by vacuum drying at 80 °C overnight. The dried coated nickel foam was pressed at a pressure of 6 MPa to form the working electrode. The counter electrode was made of platinum filament and the reference electrode was a saturated calomel. An aqueous solution of 6 M KOH was used as the electrolyte.

The electrochemical impedance spectroscopy (EIS) data was recorded (100–0.1 Hz). The gravimetric specific capacitance of the cyclic voltammetry (CV) and galvanostatic charge–discharge (GCD) curves were calculated according to Eqs. (1) and (2), respectively:

1 $$C\left( {\text{F/g}} \right) = \frac{1}{2} \times \frac{1}{m \times \Delta v \times s} \times \left( {\int\limits_{{v_{0} }}^{v} {idv} + \int\limits_{v}^{{v_{0} }} {idv} } \right)$$

2 $$C\left( {\text{F/g}} \right) = \frac{I\Delta t}{{m\Delta v}}$$

where *m* is the mass of active material (mg), Δ*v* is the potential window (V), *s* is the scan rate (mV s^−1^), *I* is the current density (mA), Δ*t* is the discharge time (s), and the integral is the area of a CV curve.

In the symmetric system, the working electrodes in the two electrodes system were prepared according to the same procedure as in the three-electrode system (described above). The aqueous solution of 6 M KOH and an organic electrolyte of 1 M 1-butyl-3-methylimidazolium tetrafluoroborate ([BMIm]BF_4_) in acetonitrile (AN) ([BMIm]BF_4_/AN) were used as the electrolytes. A piece of glassy fibrous paper (Whatman GF/G) was selected as the separator. For the aqueous electrolyte, all the components were assembled into a supercapacitor using a Swagelok type shell in air. In the case of the organic electrolyte, the supercapacitor device was fabricated in a CR2032 coin cell with Ar saturated atmosphere in a glove box to eliminate the effect of moisture and the oxygen. The two electrodes were heated in a vacuum oven at 60 °C for 24 h before use. The corresponding values for symmetric supercapacitors were calculated according to the following Eqs. (3)–(6):

3 $$C\left( {\text{F/g}} \right) = \frac{1}{m \times \Delta v \times s} \times \left( {\int\limits_{{v_{0} }}^{v} {Idv} + \int\limits_{v}^{{v_{0} }} {Idv} } \right)$$

4 $$C\left( {\text{F/g}} \right) = \frac{2I\Delta t}{{m\Delta v}}$$

5 $$E\left( {\text{Wh/kg}} \right) = \frac{{C\Delta v^{2} }}{8 \times 3.6}$$

6 $$P\left( {\text{kW/kg}} \right) = \frac{E \times 3.6}{{\Delta t}}$$

where *E* is energy density and *P* is the relative power density.

## Supplementary information

Supplementary Information
