# Supplementary material for: Eucalyptus derived heteroatom-doped hierarchical porous carbons as electrode materials in supercapacitors
Source: Sci Rep. 2020 Sep 3;10:14631. doi: 10.1038/s41598-020-71649-9 (PMC7471285; doi:10.1038/s41598-020-71649-9)
Supplement: Supplementary file 1 — Supplementary Information [file 41598_2020_71649_MOESM1_ESM.pdf]

Supporting information for

Eucalyptus Derived Heteroatoms-doped Hierarchical Porous Carbons as Electrode Materials in Supercapacitors

Yanliang Wen[1], Liang Chi[2], Karolina Wenelska[1], Xin Wen[1]*, Xuecheng Chen[1]*, and Ewa Mijowska[1]

[1]Department of Nanomaterials Physicochemistry, Faculty of Chemical Technology and Engineering, West Pomeranian University of Technology, Szczecin, Piastów Ave. 42, 71-065 Szczecin, Poland.

[2]Shanghai Institute of Space Power-sources (SISP), 2965 Dongchuan Road, Minhang District, Shanghai, China.

*Corresponding author: Xin Wen; Xuecheng Chen

Tel: 0048-914496030

Fax: 0048-914496030

E-mail: Xin.Wen@zut.edu.pl; xchen@zut.edu.pl

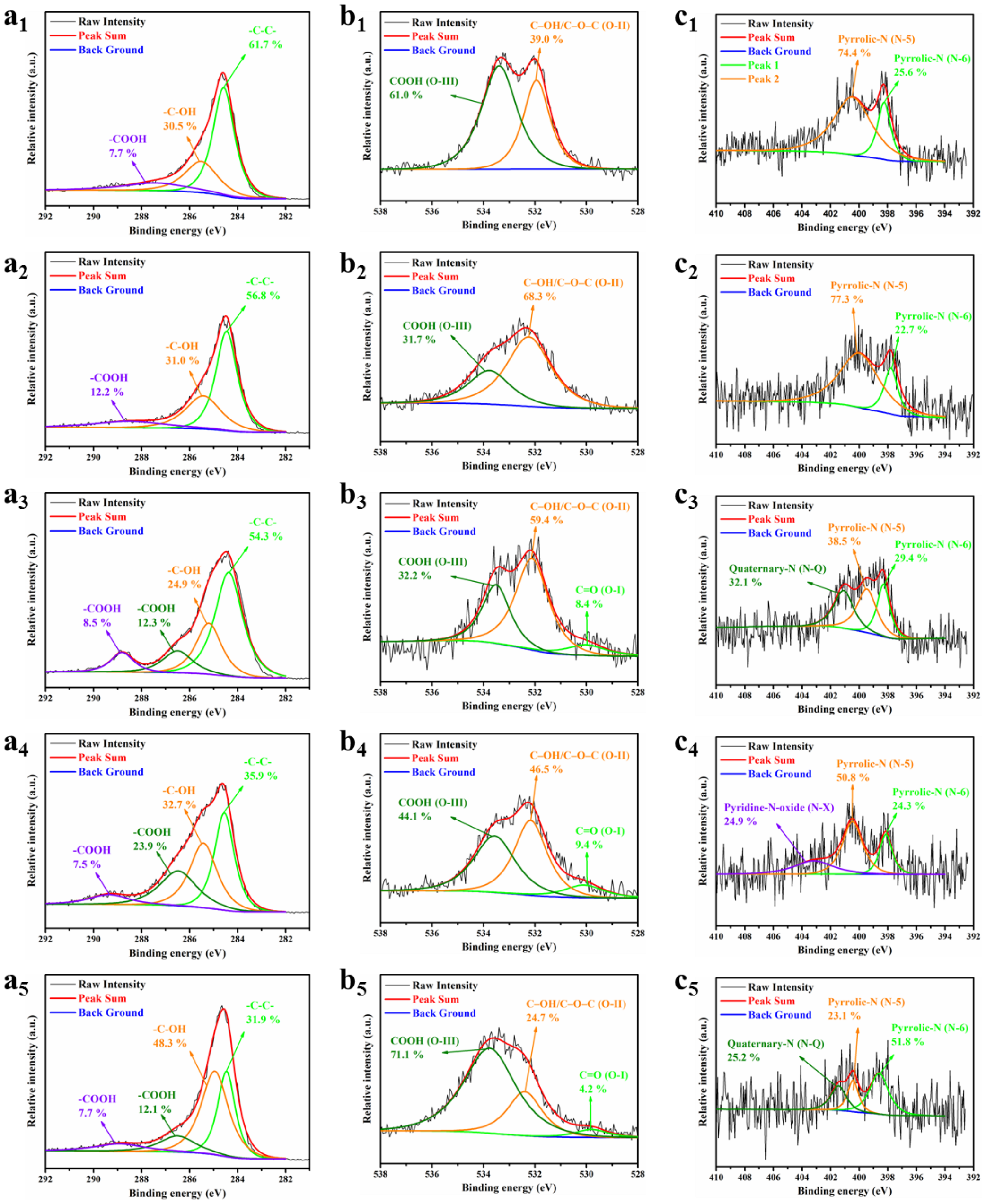

Fig. S1. High-resolution core-level XPS spectra of C1s (a_1-a_5), O1s (b_1-b_5), and N1s (c_1-c_5) for EC, NHPC-1, NHPC-3, NHPC-5, and NHPC-7, respectively.

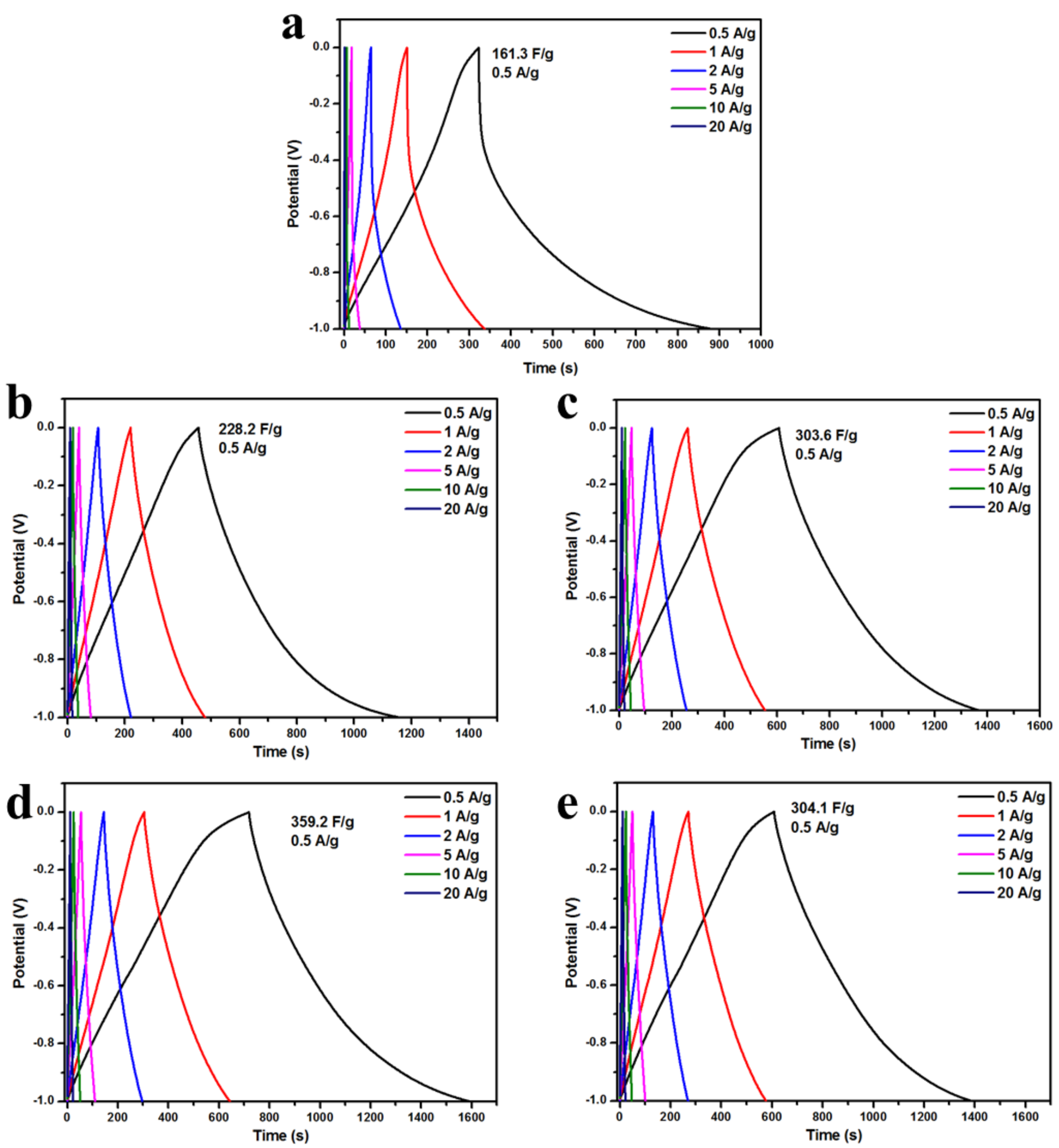

Fig. S2. GCD curves under various current densities from 0.5 to 20 A g^{-1} in a three-electrode system for (a) EC, (b) NHPC-1, (c) NHPC-3, (d) NHPC-5, and (e) NHPC-7.

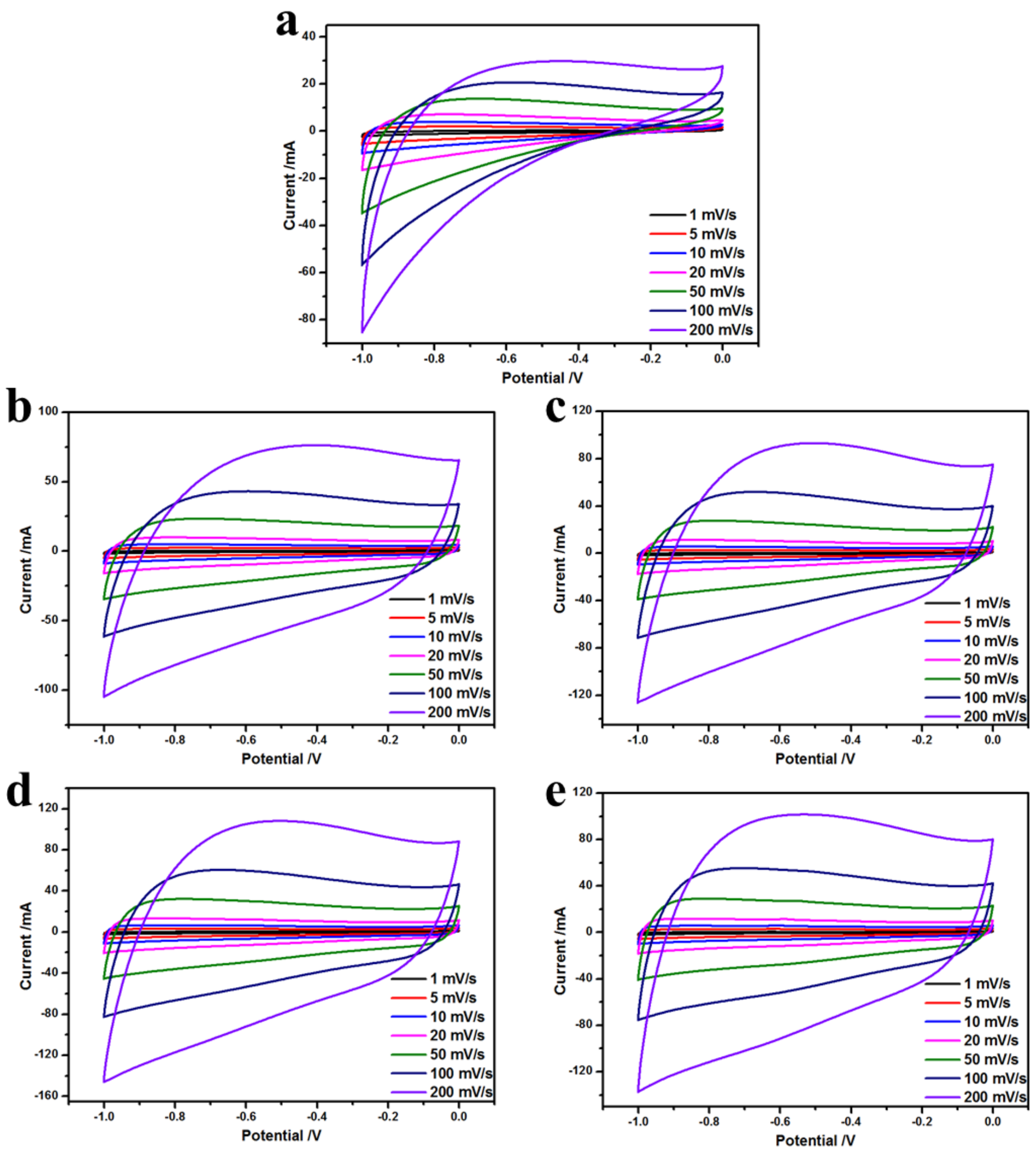

Fig. S3. CV profiles with scan rates from 1 to 200 mV s^{-1} in a three-electrode system for (a) EC, (b) NHPC-1, (c) NHPC-3, (d) NHPC-5, and (e) NHPC-7.

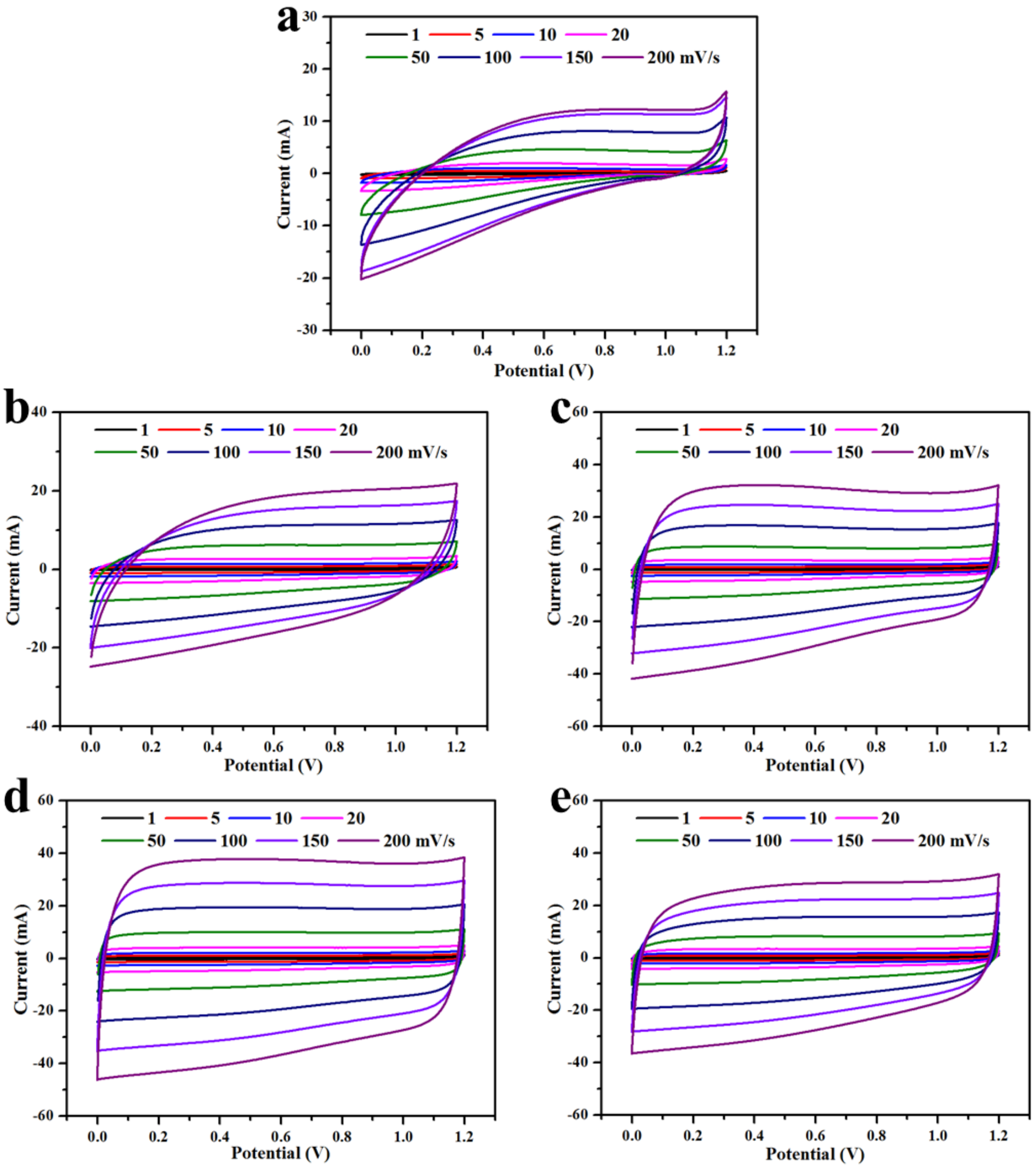

Fig. S4. CV profiles with scan rates from 1 to 200 mV s^{-1} in a two-electrode system for (a) EC, (b) NHPC-1, (c) NHPC-3, (d) NHPC-5, and (e) NHPC-7.

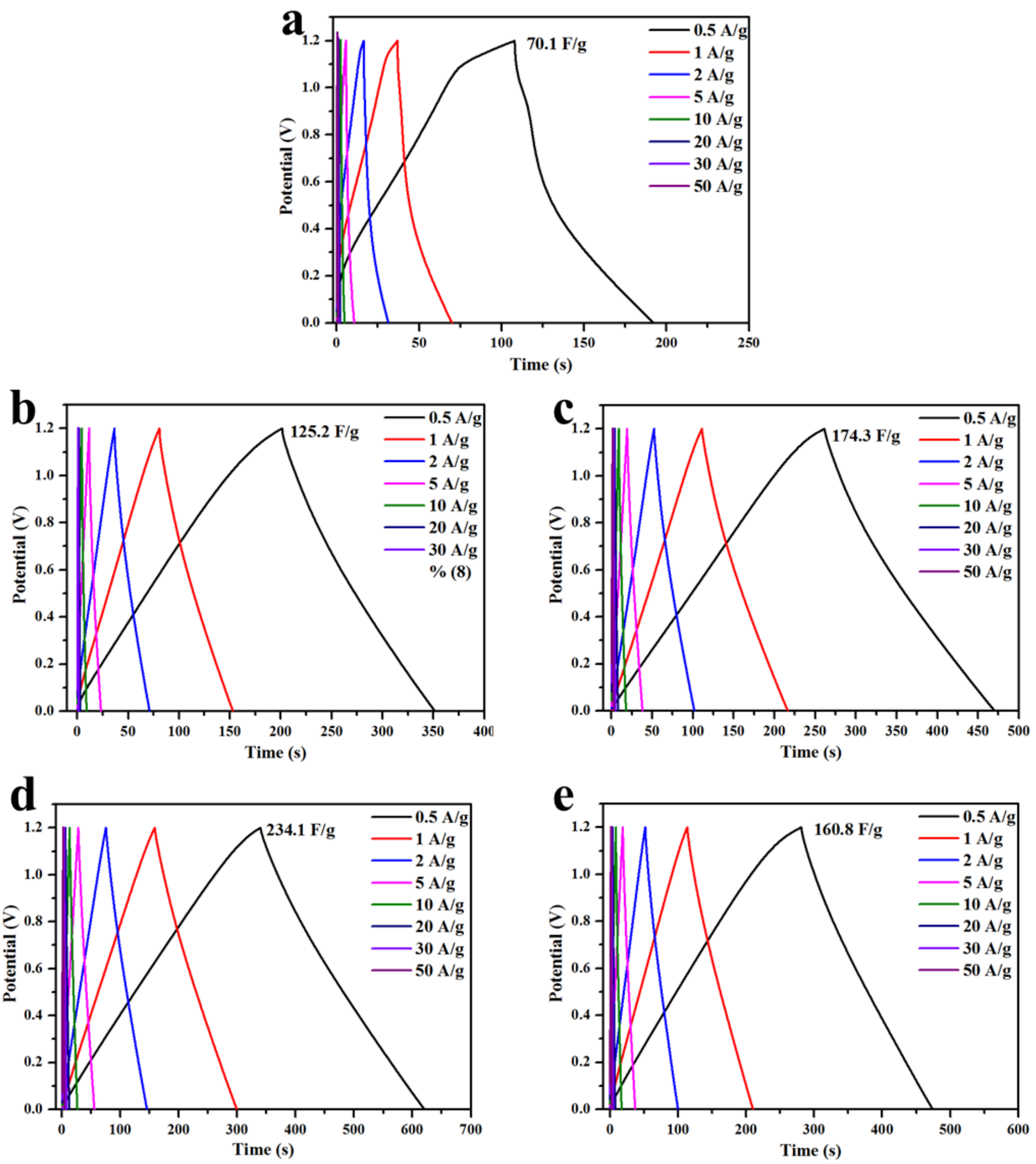

Fig. S5. GCD curves under various current densities from 0.5 to 20 A g^{-1} in a two-electrode system for (a) EC, (b) NHPC-1, (c) NHPC-3, (d) NHPC-5, and (e) NHPC-7.

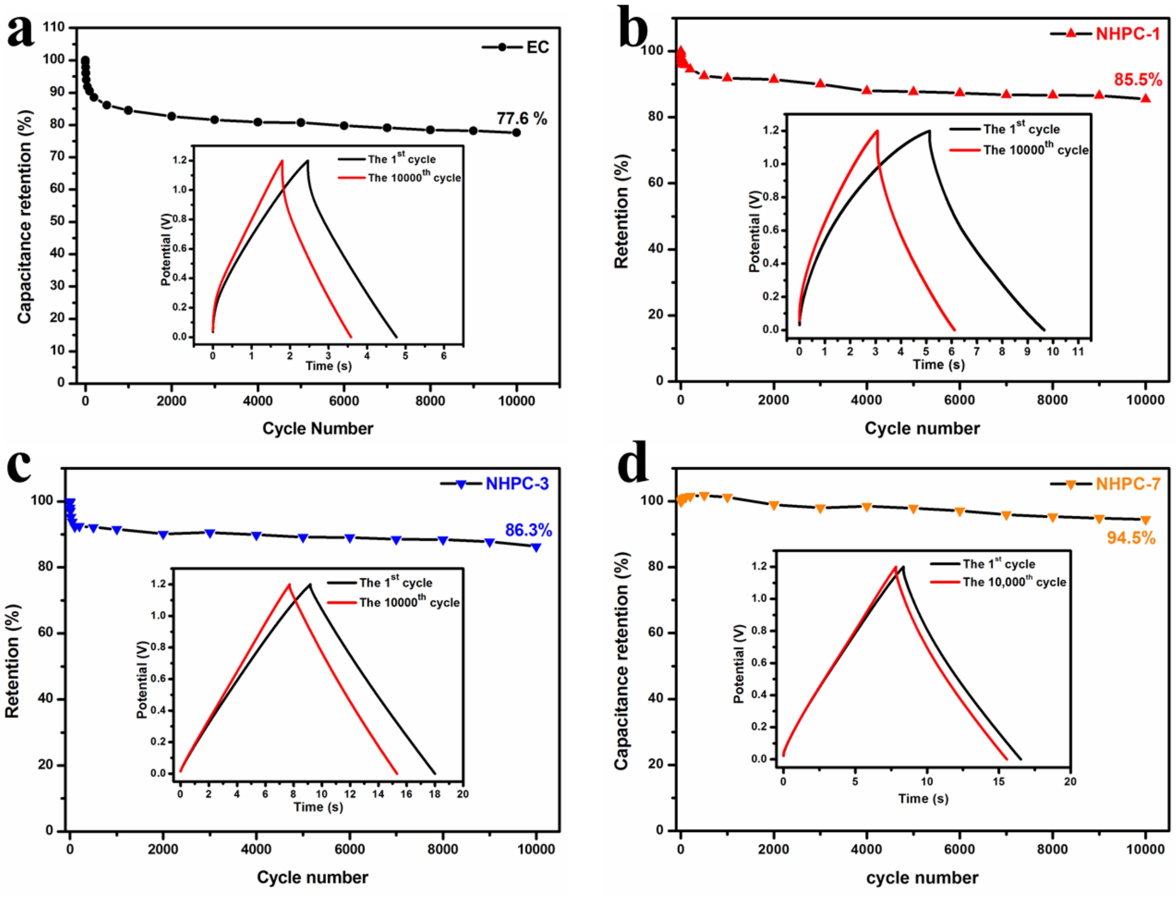

Fig. S6. The cyclic stability at 10 A g^{-1} for 10000 cycles for (a) EC, (b) NHPC-1, (c) NHPC-3, and (d) NHPC-7.

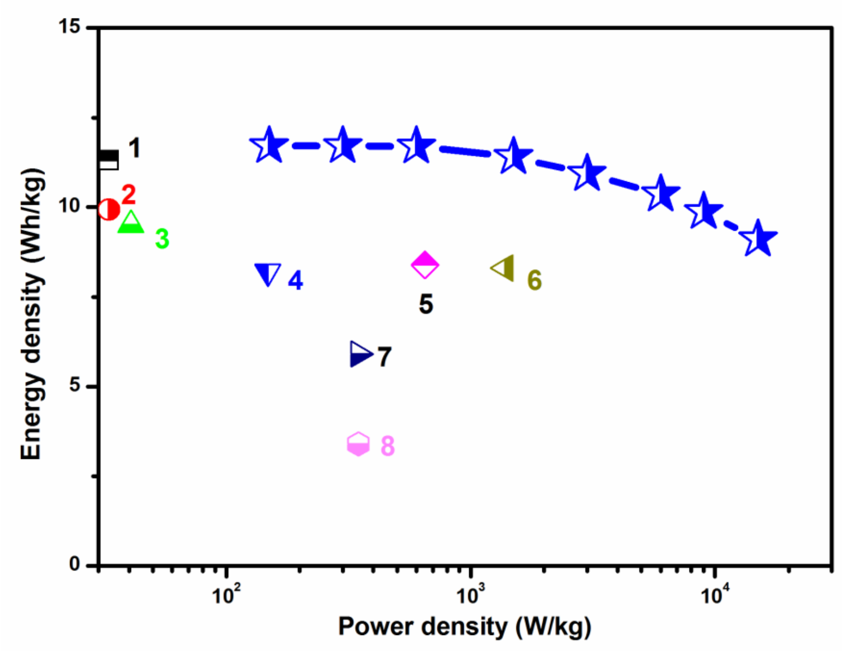

Fig. S7. Ragone plots of NHPC-5 electrode in 6M KOH and the comparison with reported works.

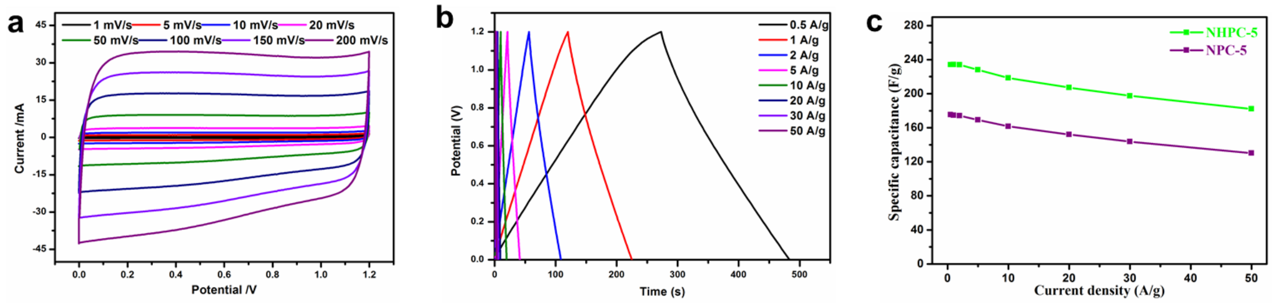

Fig. S8. (a) CV curves and (b) GCD curves of NPC-5, and (c) the capacitive comparison with the NHPC-5 electrode.

Table S1. Parameters of the peak assignment and the relative content of C1s, O1s, and N1s.

	Binding energy (eV)	Assignment	The fraction of species (%)				
			**EC**	**NHPC-1**	**NHPC-3**	**NHPC-5**	**NHPC-7**
**C1s**	284.5 ± 0.1	-C-C-	61.7	56.8	54.3	35.9	31.9
	285.3 ± 0.3	-C-O/-C-N	30.5	31.0	24.9	32.7	48.3
	286.5 ± 0.1	-C=O/-C=N	0.0	0.0	12.3	23.9	12.1
	288.7 ± 0.2	-COOH	7.7	12.2	8.5	7.5	7.7
**O1s**	530.0 ± 0.2	-C=O (O-I)	-	-	8.4	9.4	4.2
	532.1 ± 0.2	-C–OH/-C–O–C- (O-II)	39.0	68.3	59.4	46.5	24.7
	533.5 ± 0.2	-COOH (O-III)	61.0	31.7	32.2	44.1	71.1
**N1s**	398.2 ± 0.4	Pyrrolic-N (N-6)	25.6	22.7	29.4	24.3	51.8
	400.2 ± 0.2	Pyrrolic-N (N-5)	74.4	77.3	38.5	50.8	23.1
	401.2 ± 0.2	Quaternary-N (N-Q)	-	-	32.1	-	25.2
	403.2	Pyridine-N-oxide (N-X)	-	-	-	24.9	-

Table S2. Summary of the electrochemical performances of samples under different test conditions in 6M KOH.

Test Condition[a]	Capacitance (Fg^{-1})							
	3E	2E	3E	2E	3E	2E	3E	2E
	0.5Ag^{-1}		20Ag^{-1}		1mVs^{-1}		200mVs^{-1}	
**EC**	161.3	70.1	30.0	34.4	216.6	110.9	46.2	36.4
**NHPC-1**	228.2	125.2	159.4	50.1	254.6	196.5	128.8	70.9
**NHPC-3**	303.6	174.3	200.1	131.6	281.2	244.3	159.5	139.6
**NHPC-5**	359.2	234.1	230.9	207.2	327.4	264.0	186.1	172.1
**NHPC-7**	304.1	161.2	215.8	123.0	295.6	227.6	182.7	126.5

[a] 2E or 3E represents the two- or three-electrode system used in the electrochemical measurements.

Reference:

1 Liu, W.-J., Tian, K., He, Y.-R., Jiang, H. & Yu, H.-Q. High-Yield Harvest of Nanofibers/Mesoporous Carbon Composite by Pyrolysis of Waste Biomass and Its Application for High Durability Electrochemical Energy Storage. *Environ. Sci. Technol.* **48**, 13951-13959, doi:10.1021/es504184c (2014).

2 Zhang, H. *et al.* Wrinkled porous carbon nanosheets from methylnaphthalene oil for high-performance supercapacitors. *Fuel Process. Technol.* **175**, 10-16, doi:https://doi.org/10.1016/j.fuproc.2018.03.001 (2018).

3 Tian, W. *et al.* Bio-inspired beehive-like hierarchical nanoporous carbon derived from bamboo-based industrial by-product as a high performance supercapacitor electrode material. *J. Mater. Chem. A* **3**, 5656-5664, doi:10.1039/C4TA06620K (2015).

4 Shao, J. *et al.* In-situ MgO ($CaCO_3$) templating coupled with KOH activation strategy for high yield preparation of various porous carbons as supercapacitor electrode materials. *Chem. Eng. J.* **321**, 301-313, doi:http://dx.doi.org/10.1016/j.cej.2017.03.092 (2017).

5 Gao, F., Qu, J., Geng, C., Shao, G. & Wu, M. Self-templating synthesis of nitrogen-decorated hierarchical porous carbon from shrimp shell for supercapacitors. *J. Mater. Chem. A* **4**, 7445-7452, doi:10.1039/C6TA01314G (2016).

6 Cheng, P. *et al.* Hierarchically porous carbon by activation of shiitake mushroom for capacitive energy storage. *Carbon* **93**, 315-324, doi:https://doi.org/10.1016/j.carbon.2015.05.056 (2015).

7 Wen, Y. *et al.* Hierarchical porous carbon sheets derived on a MgO template for high-performance supercapacitor applications. *Nanotechnology* **30**, 295703, doi:10.1088/1361-6528/ab0ee0 (2019).

8 Wen, Y., Wen, X., Wenelska, K., Chen, X. & Mijowska, E. Novel strategy for preparation of highly porous carbon sheets derived from polystyrene for supercapacitors. *Diam. Relat. Mater.* **95**, 5-13, doi:10.1016/j.diamond.2019.03.015 (2019).
